# The Impact and Desirability of News of Risk for Schizophrenia

**DOI:** 10.1371/journal.pone.0062904

**Published:** 2013-04-29

**Authors:** Roni G. Alder, Jennifer L. Young, Elizabeth I. Russell, Danielle R. McHardy, Richard J. Linscott

**Affiliations:** 1 Department of Psychology, University of Otago, Dunedin, New Zealand; 2 Department of Psychiatry and Psychology, South Limburg Mental Health Research and Teaching Network, EURON, Maastricht University, Maastricht, The Netherlands; Maastricht University Medical Centre, The Netherlands

## Abstract

In studies of schizotypy, investigators seldom inform participants that they are engaged in research designed to shed light on risk for schizophrenia. Such nondisclosure is justified in part by the argument that disclosure of risk status may be harmful. However, there is little evidence that this is the case. Harm arising from disclosure of risk status was examined in two experiments. In the first, participants (*n* = 114 psychology undergraduates) were asked to anticipate their reactions to news of risk for schizophrenia, depression, cancer, and diabetes, and also to indicate whether they would want to know their schizophrenia risk status. Participants anticipated schizophrenia risk would have a negative impact that was significantly greater than depression or diabetes risk but similar to cancer risk. The anticipated impact of schizophrenia risk was predicted by expectations of stigmatization as well as confidence in the accuracy of biological screening. Although 81% indicated a preference for knowing their risk status, just 11% were prepared to undergo an assessment to find out. In the second, a between-subjects deception paradigm was used to inform participants (*n* = 144 psychology undergraduates) they had an enzyme deficiency that placed them at increased risk for schizophrenia, cancer, or depression. Impact was assessed using prospective self-report and salivary cortisol and retrospective self-report. Impact was modeled using measures of stigmatization and health locus of control. Retrospectively, schizophrenia, cancer, and depression risk had strong negative impacts relative to a control group, but there was no effect on prospective measures. Together, the findings suggest that news of risk for schizophrenia has the potential to engender distress, although participants’ anticipations and reflections of responses are not corroborated in prospectively measured outcomes.

## Introduction

Examinations of psychometric risk for schizophrenia are undertaken to advance understanding of schizophrenia. In such research, risk is assessed with benignly labeled measures of *personality* or *thinking* or similar, belying researchers’ interest in subclinical risk markers. In follow-up research, low- and high-risk cohorts are compared but participants themselves are not informed of their risk group status. Such nondisclosure runs against the tenet of informed consent [1] but may be warranted if the benefits of disclosure substantially outweigh its costs. Numerous factors are pertinent to the appraisal of benefits and costs of disclosure in this context: The majority of those identified will not develop a clinical disorder or need care [2], [3]; a screening assessment is an insufficient basis for judging clinical risk status; there are no services or interventions available for those exhibiting psychometric risk; the knowledge of being at risk may itself create significant psychological distress [4]; participants may place too much confidence in the predictive value of self-report screening measures; and participants may wish to reserve their right not to know their risk status. The merits of these arguments have received very little attention, either in debate or empirical examination.

In an attempt to evaluate one of these arguments, Linscott and Cross [4] asked non-help-seeking or *unsuspecting* participants to anticipate how they may respond if they were told that they were at risk for schizophrenia. Respondents rated the burden of schizophrenia risk news as greater than for heart disease risk and depression risk but less than for cancer risk. Higher anticipated burden of schizophrenia risk news was predicted by greater anxiety about risk for any disorder, greater expectation of stigmatization if diagnosed with schizophrenia, and lower psychometric risk for schizophrenia. Knowledge about schizophrenia, personal (nonpsychotic) mental health problems, and vicarious experience of psychosis were not significant predictors of the anticipated impact. We are not aware of any other directly relevant investigation of this or any other argument for nondisclosure.

Our objectives were to replicate Linscott and Cross’s [4] findings with a hypothetical paradigm and then to determine participants’ reactions when they are informed they have elevated risk for schizophrenia. Additionally, we address two important related questions that have not previously been addressed: First, do unsuspecting participants have any confidence that psychological screening accurately predicts schizophrenia risk? Secondly, do unsuspecting participants desire to know whether they are or are not at risk for schizophrenia [5], [6]?

## Study 1

Replication of Linscott and Cross [4] is essential: Findings from hypothetical paradigms are less reliable and more prone to the effects of perceptual biases; and the finding that schizotypal individuals anticipated less impact than nonschizotypal individuals was unexpected. Furthermore, Linscott and Cross [4] did not consider the potential contribution of perceived health locus of control, that is, individuals’ beliefs or expectations about determinants of health–whether health is determined by one’s own actions, the actions of professionals or important others, or the vagaries of luck [7]. Health locus of control predicts responses to and coping with both physical and psychological disorder, including psychosis [8]. In some circumstances, perceptions of control have positive effects on coping, stress, and morbidity. However, when more extreme or mismatched with disease circumstances, perceived control may have a negative effect on stress [9].

Using a similar hypothetical paradigm examining risk for schizophrenia, depression, cancer, and diabetes, we hypothesized that respondents would anticipate a high degree of burden from disclosure of risk for schizophrenia, relative to depression and diabetes. On the basis of existing research on perceptions of schizophrenia, we also hypothesized that greater burden would be associated with greater anticipation of being stigmatized because of schizophrenia [10]–[12], less knowledge about schizophrenia [13]–[15], less experience of mental health problems and less vicarious experience of psychosis [14], [16], lower schizotypy scores [2], [17], and health locus of control [9]. We expected differing proportions of participants expressing interest in knowing–in principle–outcomes of screening across the four diseases. For schizophrenia risk, we expected fewer people would commit to undergoing screening than the number expressing an in-principle interest [5].

### Methods

#### Ethics statement

The study was reviewed and approved by the University of Otago Human Ethics Committee and was conducted in accordance with the Code of Ethics for Psychologists Working in New Zealand and with APA Ethical Principles. All participants provided written informed consent to participate in the research.

#### Participants

Undergraduates (*n* = 114; age *M* = 20.4, *SD* = 4.6; 20.2% male) in introductory psychology courses volunteered as participants. The majority reported being of New Zealand European ethnicity (73.7%, Chinese = 7.0%, Maori = 5.3%, and other = 17.5%). Data from 1 person who had been diagnosed with psychosis were excluded from analysis. Following participation, participants learned about the research purpose and design; extra course credit was available on the basis of assessment of this learning, not on participation per se. Other means for obtaining extra credit (i.e., other than research participation) were also available.

#### Measures

Except for the measure of health locus of control, all measures used here were identical to those used in Linscott and Cross [4].

We assessed the anticipated impact of news, perceived screening accuracy, and preference to know risk status using a self-report measure. Participants completed the questionnaire for each disorder (schizophrenia, depression, cancer, diabetes) after being instructed to imagine their doctor informing them that, “You are at risk for [disorder]. There is a 10% chance that you will be diagnosed with [disorder] in the next 10 years.” (The scenario wording was more uniform than in the Linscott and Cross study where, for example, the scenarios made reference to *developing*, *being diagnosed with*, or *having* a disorder.).

Impact was measured using 10 items assessing felt distress, helplessness, survival expectations, coping, optimism, and impact on decision making (*How distressed would you feel? How optimistic would you be about your ability to cope with this? How anxious would you feel about your future? How likely is it that you would die from this condition? How helpless would you feel about your situation? How positive would you be about your future? How distressed would you be one week after hearing this news? How distressed would you be six months after hearing this news? How likely is it that you would be beaten by this condition? How likely are you to make different decisions about relationships and children because of this condition?*) with 7-point anchored scales (0 = *not at all*, 3 = *moderately*, and 6 = *extremely*). The schizophrenia impact score ranged from 0 = no impact to 60. We calculated a disorder-nonspecific impact score equaling the average impact for cancer, diabetes, and depression. Alpha coefficients for the impact scale ranged from α = 0.84 to α = 0.91 for specific disorders and α = 0.92 to α = 0.96 for nonspecific impact [4]. Preference for knowing risk status was assessed by asking, “If you could find out, would you want to know if you were at risk for [disorder]?”, with *yes* and *no* response options. Perceived accuracies of biological and psychological screening tests were assessed using two items (e.g., *How accurate do you think a biological screening test of risk for [disorder] would be?*) rated on a 7-point anchored scale (0 = *not accurate at all*, 3 = *moderately accurate*, and 6 = *very accurate*).

We assessed attributions of internal, powerful others, and chance health locus of control with Form A of the Multidimensional Health Locus of Control (MHLC) Scale [7]. The MHLC Scale comprises 18 items rated on 7-point anchored scales. Higher scores indicate greater perceived control by the respective agent. Reported internal consistencies and test-retest reliabilities for the MCHL subscales range from 0.60 to 0.75 and 0.60 to 0.70, respectively [18]. Observed alpha coefficients were α = 0.76 for other and chance subscales and α = 0.79 for the internal subscale.

Knowledge about schizophrenia was assessed using a 14-item multiple-choice quiz on symptoms, epidemiology, course, prognosis, treatment, etiology, and behaviour associated with schizophrenia. The observed coefficient alpha was α = 0.55, suggesting, as intended, that the quiz tapped broad range of issues. Item accuracy scores were highly correlated with those observed by Linscott and Cross [4], with *r* = 0.97, suggesting the quiz is a reliable measure.

We assessed personal and vicarious experience of mental disorder and psychosis with dichotomous (*yes*, *no*) items about: personal help-seeking for mental health problems; personal history of diagnosis of any mental disorder or psychosis; acquaintance with someone with a psychosis; regular contact with someone with a psychosis; and relatives with psychosis. Respondents were instructed that psychosis refers to a group of disorders that includes schizophrenia, schizophreniform disorder, schizotypal personality disorder, first-episode psychosis, schizoaffective disorder, and delusional disorder. Binary scores represented personal experience of any mental health problems and vicarious experience of psychosis.

Anticipated stigmatization arising from having schizophrenia was measured with the 10-item Stigma Consciousness Questionnaire (SCQ; [19]), which was designed to be adapted to different stigmatized or disadvantaged populations. Items are rated using a 7-point agreement rating scale (0 = *strongly disagree*, 3 = *neither agree nor disagree*, 6 = *strongly agree*), generating a total score ranging from 0 = no anticipated stigmatization, to 60. Alphas range from α = 0.64 to α = 0.84 and factor analyses suggest the scale is adequately described by a unitary factor [19]. The observed internal consistency was α = 0.66.

Schizotypy was assessed using the Thinking and Perceptual Style Questionnaire (TPSQ) [20], [21], a 99-item self-report measure that yields factor-based subscale scores for positive schizotypy (disordered thought, suspiciousness, hallucinatory tendency, magical thinking, perceptual aberration, self-reference ideation, and odd speech) and hypohedonia (social, emotional, gustatory, aesthetic, tactile, and exertion domains) [17]. Three composite scores were used in order not to undermine power in analyses: the sum of the positive schizotypy subscales, a social hypohedonia score (social plus emotional subscales) and a physical hypohedonia score (gustatory, aesthetic, tactile, and exertion). Unpublished data show these three composite scores have good test-retest reliability (*r* = 0.84, *r* = 0.78, and *r* = 0.76, respectively; *n* = 284) and satisfactory internal consistency (α = 0.92, α = 0.79, and α = 0.68, respectively; *n* = 1543).

The in-principle preference for knowing one’s risk status may be more readily expressed than the willingness complete an assessment. The latter was assessed for schizophrenia risk using a written invitation with a *yes* and *no* response option:

*Taking part in this study may have prompted you to think about whether you are at risk for schizophrenia. We would like to know if you would be interested in arranging an appointment to undergo psychological screening for risk for schizophrenia. Note: A [licensed] clinical psychologist would conduct the screening. Screening would be offered as a free confidential service and held at a time that is convenient to you. Information collected would not be disclosed to others except at your request. Would you like to arrange an appointment?*

Following participation, participants were debriefed as to the purpose of this question and informed that a screening service was in fact not available. Participants were also informed that if the study raised any concerns about their mental health, that advice could be sought from the university’s health and counseling service, who had been informed about the study.

#### Statistical analyses

Data are available from the corresponding author. Kurtosis and skewness of key dependent measures were examined using sktest and boxcox functions in Stata 10.1. Screening accuracy ratings required Box-Cox transformation whereas other measures did not depart from normality. Univariate outliers were detected by histogram, and multivariate outliers by leverage and Cook’s distance. There was no evidence of outliers. Differences in the impact of disorders were assessed using within-subjects repeated measures analysis of variance (ANOVA). Schizophrenia impact was modeled with linear regression. Analyses were performed using PASW Statistics 18.0 and Stata 10.1.

### Results

#### Burden across disorder

**Figure 1:**
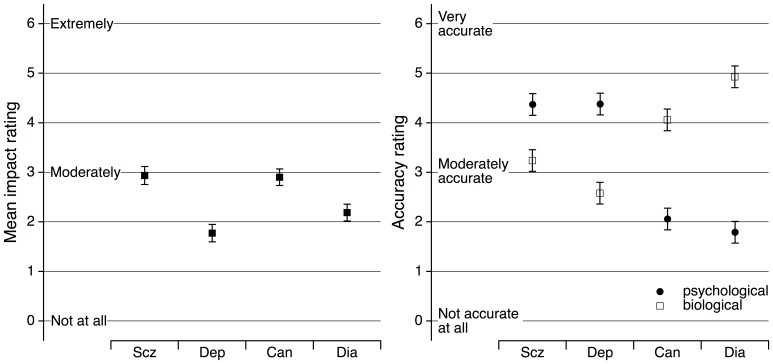
Impact and accuracy ratings obtained in Study 1. Left panel. Mean item rating for anticipated adverse impact of news of risk about schizophrenia (Scz), depression (Dep), cancer (Can), and diabetes (Dia). Right panel. Mean ratings of the perceived accuracy of psychological and biological screening for the four disorders. All error bars represent 95% confidence intervals.

The anticipated adverse impact of risk news varied across disorder, *F*(3, 336) = 87.2, *MSE* = 41.7, *p*<.001, η^2^ = .44 (Figure 1). All planned contrasts showed significant disorder differences (*p*<.001, with *d* = 0.48 to 1.28) except for that between schizophrenia and cancer, where *t*(112) = 0.39, *p* = .88, *d* = 0.04. Schizophrenia and cancer were associated with the greatest impact followed by diabetes and depression.

#### Perceived accuracy of screening

A 2×4 within-subjects ANOVA (screening mode×disorder) of perceived accuracy ratings yielded a significant interaction of mode and disorder, *F*(3, 336) = 234.8, *MSE* = 2.14, *p*<.001, η^2^ = .68, and main effects for mode and disorder, with *F*(1, 112) = 37.0, *MSE* = 2.67, *p*<.001, η^2^ = .25, and *F*(3, 336) = 19.8, *MSE* = 1.60, *p*<.001, η^2^ = .15, respectively (Figure 1). Considering just those disorder ratings within the modes judged most accurate for the respective disorder, perceived accuracies were least for cancer, followed by schizophrenia, depression, and diabetes. Pairwise contrasts were all significant (*p*≤.022), except for the comparison of depression and schizophrenia, *t*(112) = 0.12, *p* = .91, *d* = 0.01. Participants perceived psychological screening for risk of schizophrenia as more accurate than biological screening for risk of cancer, *t*(112) = 2.32, *p* = .022, *d* = 0.22.

#### Regression models of schizophrenia impact

Two regression models of schizophrenia impact were examined. In Model 1, schizophrenia impact was regressed onto nonspecific impact, stigma, and positive schizotypy, the model reported by Linscott and Cross [4]. Model 1 was significant but positive schizotypy was not a significant predictor (Table 1). Beta coefficients obtained for nonspecific impact and stigma did not differ from those reported by Linscott and Cross, with *F*(2, 109) <1.

**Table 1:** Regression models of schizophrenia impact.

		Model 1	Model 2	
Predictor		ß	Δ*R* ^2^	ß
Nonspecific impact		.62***		
Stigma		.25***		
Positive schizotypy		.07		
Step 1			.39***	
	Nonspecific impact			.62***
Step 2			.06***	
	Nonspecific impact			.64***
	Stigma			.25***
Step 3			.03*	
	Nonspecific impact			.59***
	Stigma			.25***
	Screen accuracy, biological			.18*
Total adjusted *R* ^2^		.46		.48
*n*		113		113
*F*		30.55***		33.97***

* *p*<.05.

*** *p*<.001.

Model 2 was developed using stepwise regression of schizophrenia impact on to 13 predictors: nonspecific impact, three locus of control indices, physical and social hypohedonia, positive schizotypy, knowledge, personal and vicarious experience (coded 0 or 1), stigma, and perceived accuracy of biological and psychological screening methods. The entry and removal criteria were *p*<0.05 and *p*>0.10, respectively. The analysis concluded after entry of nonspecific impact, stigma, and the perceived accuracy of biological screening for schizophrenia risk (Table 1). Model 2 was significantly better than Model 1, *F*(1, 109) = 14.01, *p*<.001. Adding sex and minority ethnic status did not affect the outcome of the results.

A control analysis was undertaken by regressing the cancer impact score on to the same set of predictors (except, the nonspecific impact was the average of the diabetes and depression impact). This analysis concluded after one step in which nonspecific impact was entered (*B* = 0.61, *SE B* = 0.08, ß = .57), giving *F*(1, 111) = 52.05, *p*<.001, *r* ^2^ = .319.

#### Preference and willingness to know screening outcomes

Preference for knowing screening outcomes was associated with disorder, log likelihood χ^2^(3, *N* = 448) = 35.1, *p*<.001. More participants preferred to know about risk for schizophrenia (81.1%, *n* = 111) than risk for depression (67.9%, *n* = 112), Yates’ corrected log likelihood χ^2^(1, *N* = 223) = 4.49, *p* = .034, and more favored knowing about diabetes risk (96.4%, *n* = 112) than cancer risk (81.4%, *n* = 113), Yates’ corrected log likelihood χ^2^(1, *N* = 225) = 12.2, *p*<.001. The numbers who preferred to know about risk for cancer and risk for schizophrenia did not differ, Yates’ corrected log likelihood χ^2^(1, *N* = 224) = 0.01. Preference for knowledge of risk was strongly correlated across the four disorders, with ρ = .61 to ρ = .82. By comparison, when participants were given the opportunity to make an appointment to undergo screening for risk for schizophrenia, just 11.1% (of *n* = 108) were willing to take up the offer. All of these were people also responded affirmatively to the preference question.

### Discussion

The anticipated impacts of knowledge of risk for schizophrenia and cancer were on par. Schizophrenia impact was best accounted for by a regression model including three predictors: Greater impact was predicted by higher anticipated stigmatization, greater confidence in the accuracy of biological screening for schizophrenia risk, and higher nonspecific impact. Indices measuring hypohedonia, personal experience of mental health problems, health locus of control, knowledge about schizophrenia, and schizotypy were not significant predictors of schizophrenia impact. Participants believed that psychological measures that screen for risk for psychological disorders, and biological screens for risk for physical disorders, were mid way between *moderately* and *very* accurate. Moreover, psychological indices of schizophrenia risk were rated as more accurate than biological indices of cancer risk.

Whereas four in five people indicated that, if it was possible to know, they would like to know their schizophrenia risk status, only one in ten accepted an invitation to undergo screening. Surprisingly, fewer people, two in three, indicated a preference to know their depression risk status.

## Study 2

It is unclear to what degree the findings derived using hypothetical paradigms portray what would happen if unsuspecting research participants were told they are at risk for schizophrenia. The brevity of the procedure in Study 1 and the lack of any need or opportunity to reconcile a hypothetical risk status with current or future life goals, quality of life, and other relevant variables, create uncertainty about the generalizability of the findings. Judgments of anticipated behaviour are not necessarily predictive of actual behaviour and may be influenced by availability heuristics, perceptual biases, and social desirability [22].

Given these limitations, the aim of Study 2 was to determine whether the actual impact of risk news was consistent with Study 1 findings. Given uncertainty about the repercussions of risk news, we elected to use the thioamine acetylase enzyme deficiency (TAED) deception paradigm [23], [24]. In this paradigm, volunteers are screened for a benign enzyme deficiency that is presented to them as a known risk factor for a disorder of interest. The screening test, which is rigged to provide a positive result, involves reagent testing of saliva obtained using a mouthwash procedure. Thus, the inevitably positive test result conveys implicitly to participants that they are also at risk for the disorder of interest. At the completion of assessment, volunteers learn that TAED is a bogus condition and that the TAED screening test is rigged. This paradigm has two important qualities. First, the procedure allows unconfounded comparisons of any number or combination of disorders of interest. Secondly, once assessment and debriefing are completed, there are no objective residual grounds upon which a participant may believe that the alleged elevated risk is nevertheless genuine.

In Study 2, we compared the acute effects of news of risk for schizophrenia, cancer, or depression on salivary cortisol and state mood, testing several hypotheses. First, change in objective and subjective stress indices will show that risk for schizophrenia has an impact that is greater than risk for depression and membership in a TAED-only control group, but similar to risk for cancer. Second, anticipated stigmatization and health locus of control will influence the stress reaction elicited by risk news. Third, having been exposed to the stress procedure and then debriefed, retrospective evaluation of the impact of the risk news will be greater in the schizophrenia and cancer groups than in the depression and control groups.

### Methods

#### Ethics statement

The study was reviewed and approved by the University of Otago Human Ethics Committee and was conducted in accordance with the Code of Ethics for Psychologists Working in New Zealand and with APA Ethical Principles. All participants provided written informed consent to participate in the research. This study involved deception. The deception was justified to the University of Otago Human Ethics Committee and the procedure was executed in a manner consistent with the aforementioned ethical codes. A condition of ethical approval was that the Committee closely monitored the outcome observed for first ten participants and, subsequently, as required.

#### Participants

Undergraduate students (*n* = 168, age *M* = 21.4, *SD* = 4.2; 54% male) enrolled in second-year psychology courses volunteered as participants. Most identified as New Zealand European (70%; Chinese, 5%; Indian, 5%; Maori, 5%; Pasifika, 2%; others, 13%). Participants were randomly allocated to one of the four groups (control, schizophrenia, depression, cancer).

Inclusion criteria included fluency in English, 17 years or older, no current or previous psychological disorder, no history of cancer, and no family history of psychosis. Eight participants were excluded (*M* = 28.0, *SD* = 13.1 years; 4 males) for one or more reasons including a history of or current anxiety disorder (*n* = 7) or depression (*n* = 7), current or past paranoia (*n* = 2), a family history of mental disorder (*n* = 4), and a history of cancer (*n* = 1). All participants–including those not meeting inclusion criteria–were eligible to receive a small amount of course credit by completing a brief worksheet about research design.

#### Measures

Measures included the 10-item impact questionnaire, MHLC, and the SCQ described in Study 1. Item wording in the impact questionnaire was modified to correct tense (e.g., “How distressed would you feel?” became “How distressed did you feel?”). In the SCQ, the focus was on the presence of a serious physical or mental disability (cf. *schizophrenia* in Study 1).

The Profile of Mood States (POMS; [25]) was used as a measure of subjective mood state. The POMS is a self-report adjective rating scale comprising 65 items with 5-point Likert scales (0 = *not at all*, 1 = *a little*, 2 = *moderately*, 3 = *quite a bit*, 4 = *extremely*), yielding scores on six subscales: depression-dejection, tension-anxiety, confusion-bewilderment, anger-hostility, fatigue-inertia, and vigor-activity. Higher scores indicate higher levels of the corresponding construct. Subscale alphas range from α = .84 to.95. POMS scores are sensitive to moment-to-moment changes in mood and the instrument is appropriate for use with undergraduates [25]. Two scores were used: the sum of the 5 negative affect subscales and the vigor score.

The screening and psychotic screening modules from the *Structured Clinical Interview for DSM-IV* (SCID-I; [26]) were used to screen for present and past episodes of psychopathology. Together these consist of 20 items that allow for follow-up questions to probe the extent and impact of any problem identified by the respondent. Four supplementary questions on the history of cancer, psychosis, depression, and family history of mental illness were added to the modules.

A 6-item cortisol survey was used to assess respondents’ exposure to non-experimental factors that affect cortisol (amount and recency of sleep, Vitamin C and caffeine consumption, use of contraceptives and cigarettes). Item content was derived from the literature on cortisol [27]–[33]. Each question had a dichotomous YES-NO response format and allowed for probing for additional information as required.

#### Salivary cortisol

Passive drool samples (≥1 ml) were obtained using short plastic straws and 5 ml sample tubes and frozen until assay. First samples were obtained between 1200 and 1530 hours for all participants. Samples were stored at −20°C until required for analysis. Samples were thawed and centrifuged at 3000 rpm for 15 min and 250 µl of saliva was obtained from each sample. Saliva was extracted with 1.0 ml dichloromethane and vortexed for 2 min and 500 µl was dried. Samples were reconstituted with 125 µl assay buffer for enzyme-linked immunosorbent assay (ELISA). Recovery of cortisol over the operating range is 90–100% using spiked saliva; recovery using 3H-cortisol is 102%. Within assay variation is less than 20%. ELISA is suited to diurnal variation studies and experimental research. More details on the ELISA method and validation are reported elsewhere [34]–[37].

#### TAED diagnostic test

Participants rinse their mouths with water and, after 5 seconds, expel the water into a sample cup. A commercial test strip is then dipped in the cup and left in front of the participant while the experimenter and participant continue with other tasks. Unknown to the participant, the mouth rinse contains both water and glucose (∼2.9%), at a concentration that ensures the glucose is tasteless, and the test strip is a glucose test pad from a reagent strip. Thus, the test procedure always gives a positive TAED test result. We trimmed Siemens Multistix 8 SG or 10 SG reagent strips for urinalysis on which the glucose test pad is adjacent to the hold area.

#### Procedure

Following random assignment to experimental groups, participants received group-specific information sheets containing bogus information about TAED and, as applicable, its relevance to risk for schizophrenia, cancer, or depression. After consent was obtained, participants rinsed their mouths with water before completing the POMS. The researcher then casually repeated some of the detail contained in the information sheet, including its link “with a ten-fold increase in risk for [depression/schizophrenia/cancer]” according to group membership. Participants were informed that TAED itself was asymptomatic and benign.

Inclusion criteria were assessed with the SCID-I screening modules. Participants who met inclusion criteria completed the cortisol survey and provided a baseline saliva sample. The TAED diagnostic test was described and participants were informed that a positive test result was unlikely as TAED affected just 1 in 15 people. When the test strip gave a positive result, the experimenter expressed mild surprise and invited the participant to complete a neuropsychological assessment of semantic memory [38] consistent with the ostensible purpose of the study. This assessment provided time both for participants to think about the TAED result and for a cortisol response. After 25 minutes, the participant completed the post-stress assessment, which included the POMS, collection of saliva, the MHLC, and the SCQ.

Researchers then fully debriefed participants on the fictitious nature of the TAED test, the true purpose of the study, and the study rationale. (Those not meeting inclusion criteria were also fully debriefed.) Participants saw how the TAED diagnostic test was rigged so that positive results were inevitable. Participants were then asked to indicate whether they had believed TAED was a real condition and to complete the 10-item impact questionnaire. Throughout the procedure, researchers noted observed changes in participants’ behaviour.

Researchers were not blind to the study purpose or group assignment. Participants were assessed between 12 pm and 4 pm in order to limit the effects of circadian variation on salivary cortisol [39]–[41].

#### Analyses

Data are available from the corresponding author. Kurtosis and skewness were examined using Stata 10.1 sktest and boxcox functions, univariate outliers were detected using histograms, and multivariate outliers were identified using leverage and Cook’s distance. Box-Cox transformation was applied to correct non-normal baseline and post-manipulation cortisol and negative POMS indices, and the retrospective impact score. Transformed cortisol and POMS scores were standardized to permit comparison. Baseline cortisol was regressed onto cortisol survey responses and the residuals were obtained, giving baseline cortisol corrected for measured sources of error. The regression model obtained using baseline cortisol was then applied to post-manipulation cortisol to obtain its residual.

Multiple regression was used to test hypotheses. Regression modeling of post-manipulation cortisol and POMS each proceeded through three steps. At Step 1, the corresponding baseline measure was the sole predictor. This served to minimize the effects of individual differences, particularly in salivary cortisol and exposure to uncontrolled variables that affect cortisol (e.g., menstrual cycle, although see [40]). At Step 2, group membership dummy variables were added; and at Step 3, SCQ and MHLC variables were added. The power to detect a medium effect of group on post-manipulation cortisol and POMS was 0.98 with α = .05. Retrospective impact ratings were regressed onto baseline cortisol and POMS measures at Step 1, dummy variables for group membership were added at Step 2, and SCQ and MHLC were added at Step 3 (power = 0.97 with α = .05).

### Results

Data from 2 multivariate outliers and 16 participants who reported not believing TAED was a genuine condition were removed from further analysis, giving a final *n* = 142. Technical problems caused the loss of cortisol samples and data from another 6 participants; in analyses involving cortisol, *n* = 136. Descriptive statistics are reported in Table 2.

**Table 2:** Subjective and objective measures of distress at baseline and post-manipulation.

	Control		Schizophrenia		Cancer		Depression	
	*M*	*SD*	*M*	*SD*	*M*	*SD*	*M*	*SD*
	(*n* = 38)		(*n* = 34)		(*n* = 32)		(*n* = 38)	
Baseline								
Negative affect	26.5	20.8	23.1	13.8	26.4	21.0	27.7	24.2
Vigor	15.6	4.4	15.1	5.5	14.4	5.5	14.0	5.4
Cortisol^a^	16.2	6.3	30.1	56.2	16.7	10.0	23.9	32.9
Post-manipulation								
Negative affect	21.1	16.1	21.3	16.4	25.0	20.6	24.0	19.9
Vigor	12.8	4.4	12.0	5.3	11.0	5.7	11.0	5.9
Cortisol^a^	16.1	10.4	22.6	31.5	14.8	7.3	22.8	26.0

a For cortisol, *n* = 37, 33, 30, and 37, for the four groups, respectively.

#### Regression of prospective and retrospective impact

There was no evidence the cortisol and POMS outcomes were affected by the group manipulation, SCQ, or MHLC variables (Table 3). In modeling each outcome variable, only baseline measures of the outcome variable were significant predictors. Beta coefficients for the schizophrenia, cancer, and depression groups did not differ in any final model, with all *t* <1.0. Moreover, no model had the expected pattern of group effects, namely that schizophrenia and cancer risk news would be more adverse than the depression risk news.

**Table 3:** Regression weights for predictors of post-manipulation cortisol, negative mood, and vigor.

		Post-manipulation outcome					
Predictor		Cortisol		POMS negative		POMS vigor	
		Δ*R* ^2^	ß	Δ*R* ^2^	ß	Δ*R* ^2^	ß
Step 1		.53***		.67***		.59***	
1	Baseline^a^		.73***		.82***		.77***
Step 2		.01		.01		.00	
2	Baseline^a^		.73***		.82***		.76***
	Schizophrenia		.06		.00		−.04
	Cancer		−.00		.09		−.07
	Depression		.10		.04		−.05
Step 3		.01		.02		.01	
3	Baseline^a^		.72***		.80***		.76***
	Schizophrenia		.06		−.01		−.03
	Cancer		.01		.05		−.05
	Depression		.10		.03		−.04
	SCQ		−.06		.13*		−.06
	MHLC, Internal		.02		−.00		−.04
	MHLC, Other		−.01		.02		−.02
	MHLC, Chance		−.04		.03		−.01
Total adjusted *R* ^2^		.52		.67		.57	
*n*		136		142		142	
*F*		19.05***		37.13***		24.56***	

MHLC = Multidimensional Health Locus of Control; POMS = Profile of Mood States; SCQ = Stigma Consciousness Questionnaire.

a The baseline value of the corresponding outcome variable.

* *p*<.05.

*** *p*<.001.

Retrospective impact ratings were affected by group membership, which produced medium to large effects, with *f* ^2^ = 0.27 (Table 4). However, the magnitude of the effects did not differ across groups, with *t* <1.0 in each contrast. Impact was predicted by greater baseline negative POMS and powerful others MHLC. In contrast, baseline cortisol, SCQ, and internal or chance MHLC scores were not significant predictors of impact.

**Table 4:** Regression models of retrospective impact.

Predictor		Δ*R* ^2^	ß
Step 1		.12***	
	Baseline cortisol		−.14
	Baseline POMS negative		.32***
	Baseline POMS vigor		.09
Step 2		.17***	
	Baseline cortisol		−.12
	Baseline POMS negative		.34***
	Baseline POMS vigor		.14
	Schizophrenia risk news		.39***
	Cancer risk news		.35***
	Depression risk news		.43***
Step 3		.08**	
	Baseline cortisol		−.13
	Baseline POMS negative		.31***
	Baseline POMS vigor		.15
	Schizophrenia risk news		.33***
	Cancer risk news		.33***
	Depression risk news		.37***
	Stigma consciousness		.08
	MHLC, Internal		−.05
	MHLC, Other		.27***
	MHLC, Chance		.03
Total adjusted *R* ^2^		.32	
*n*		136	
*F*		7.32***	

* *p*<.05.

** *p*<.01.

*** *p*<.001.

Adding sex and minority ethnic status to regression models for prospective and retrospective outcomes did not affect findings. Also, across all regression analyses, tolerance coefficients were ≥0.65, indicating no evidence of multicollinearity.

#### Researchers’ observations

Following the TAED diagnostic procedure, researchers noted some participants grew more anxious or worried looking, frowned, blushed, appeared surprised, or laughed nervously. Some tensed up, clenching hands or biting nails. Some became quiet or more serious. Some appeared indifferent or unaffected. Many participants engaged in information gathering behaviour, or remarked they would. Participants double-checked the test strip, re-read the information sheet, asked about TAED, commented about “Googling TAED”, or asked questions such as about the relationship of TAED to other conditions (e.g., anemia) or the meaning of terms (e.g., benign). Some discussed referral to a physician. Some participants remarked on the TAED result, saying things such as “That’s not good news”, attributing the result to stress, and describing risk for schizophrenia as “scary”. A participant in the control group remarked that her TAED-positive status was “Another thing to add to the list.” Surprisingly, on debriefing, one participant in the schizophrenia group expressed disappointment, saying that she wanted to tell people she was at risk for schizophrenia.

### Discussion

There was no prospective evidence that risk news was stressful. In contrast, risk news was appraised in retrospective ratings as more adverse than the TAED-only status but the degrees of adversity associated with cancer, depression, and schizophrenia did not differ significantly. Baseline negative mood and confidence in professionals’ ability to determine health outcomes were important predictors of retrospective impact ratings. Therefore, findings were not consistent with our expectations: Schizophrenia risk news was not more adverse than that for depression or TAED-only news, and impact was not a function of anticipated stigmatization or health locus of control. Also, participants’ retrospective reactions to risk appeared not to distinguish disorders.

Several factors affect the interpretation of these findings. Without a non-TAED control condition, we cannot test our assumption that the TAED manipulation itself would have no adverse effect, although the group effect on retrospective ratings suggests the assumption is not unreasonable. At 25 minutes, the risk news exposure period was quite brief, albeit sufficient in duration to allow detectable changes in mood and salivary cortisol [42]. Others have also reported the relationships of subjective distress and exposure to stressful events with salivary cortisol are dissociable [43]. Moreover, participants were excluded from the analyses if they did not believe the deception. Importantly, the study was adequately powered to detect a medium effect of group on prospective outcomes in mood and cortisol–effects considerably smaller in size than those observed in Study 1. The assessment methodology did not correspond to that used in schizotypy research. Finally, the risk news was conveyed through remarks that were constructed as coincidental in the context, not delivered in a manner consistent with a screening programme.

## General Discussion

Participants’ anticipations and reflections suggested that schizophrenia risk news was perceived as adverse but these perceptions were not corroborated by prospective stress measures. Moreover, differences between anticipated and retrospective judgments may imply that impact evaluations were not driven by schizophrenia risk per se but by appraisals of the health-related threat created through the study paradigm. When risks for different types of outcomes were contrasted (Study 1), stigma was an important basis for judging impact. However, when risks were evaluated in isolation (Study 2), anticipated stigma had little or no effect. Instead, the perceived impact was a function of prevailing mood and expectations about the power of professionals to influence health outcomes. Finally, of those who indicated they would like to know their schizophrenia risk status, very few were prepared to invest time in finding out.

For other health conditions, family history and experience of prodromal, subclinical, or mild symptoms of disorder have been found to affect reactions to screening outcomes and the desire to obtain risk information. For genetic or familial conditions, positive screening results do not necessarily engender distress [44]–[46]. For those undergoing genetic testing, screening generally leads to reduced distress regardless of the outcome, possibly because screening reduces uncertainty [44] or because of the influence of pre-screening expectations [47]. In such circumstances, positive test results predict a slower and less pronounced reduction in distress than negative test results [44]. As was found with the retrospective ratings in Study 2, distress following genetic screening appears to be more strongly predicted by pre-screening mental state than by the screening outcome itself [44]. Thus, the mental health and psychosis related inclusion criteria in Study 2, which were applied for ethical reasons, might have introduced an important bias in the sample.

The negative effects of screening appear to be related to the sense of threat or vulnerability that arises from a positive result rather than the specific disease. Such effects mostly appear to be relatively short-lived and not detectable beyond a month following screening [45]. However, studies of screening for Huntington’s disease show that positive results can predict extreme negative outcomes (e.g., suicidal behaviour and psychiatric hospitalization) in the 12 months following screening [46]. Extreme negative outcomes were also predicted by psychiatric history, unemployment, and recent diagnosis or the onset of symptoms [46]. Whether findings from the ∼30 minute controlled exposure to risk news in Study 2 generalize to a longer or uncontrolled exposures is unclear.

Of course, screening for psychometric risk for schizophrenia does not occur in the same manner as screening for Huntington’s disease or other familial or genetic conditions. For the latter, individuals self-select because of family history and, consequently, may be better equipped to deal with risk news than those who do not self-select [44] or may be anticipating positive screening results and so react less negatively when such results are realized [47]. In contrast, participants in psychometric risk research are neither self-selected nor anticipating a risk outcome. A large number of other factors also distinguish participants in psychometric risk research from those seeking screening for familial or genetic conditions: Psychometric risk research is almost completely removed from clinical practice; research participants are not help-seekers or expecting clinical benefits but are be motivated by other factors (e.g., altruism, monetary or academic rewards, learning objectives); the participant–investigator relationship is unlike the patient–clinician relationship (e.g., the investigator is the principal beneficiary, investigators initiate and terminate the relationship); research protocols are rigid and largely unresponsive to participants’ health needs; research tasks and procedures need not meet standards for clinical use; and individual–level data seldom have clear clinical meaning.

In summary, news of risk for schizophrenia had a measureable subjective impact on unsuspecting research participants, albeit an impact that was not reflected in ratings of affect or in salivary cortisol. In line with research into clinical screening for familial and genetic conditions, the impact appeared not to be specific to schizophrenia but may be understood as arising from a sense of threat or vulnerability arising from this risk news. The impact was also strongly related to baseline mood. These findings suggest there is some merit in arguing that disclosure of psychometric risk for schizophrenia may itself be risky, particularly given a majority of those at risk are unlikely to experience psychiatric morbidity [3] and the relatively high level of confidence that is placed in the accuracy of psychological screening. If strategies for disclosure are considered, these should accommodate the needs of individuals who wish not to know their risk status.
